# Invasion and Metastasis as a Central Hallmark of Breast Cancer

**DOI:** 10.3390/jcm10163498

**Published:** 2021-08-08

**Authors:** Trishna Saha, Jonathan Solomon, Abraham O. Samson, Hava Gil-Henn

**Affiliations:** 1 Cell Migration and Invasion Laboratory, The Azrieli Faculty of Medicine, Bar-Ilan University, Safed 1311502, Israel; trishna.saha27@gmail.com (T.S.); yhontn@gmail.com (J.S.); 2 Drug Discovery Laboratory, The Azrieli Faculty of Medicine, Bar-Ilan University, Safed 1311502, Israel

**Keywords:** breast cancer, hallmarks, invasion and metastasis, therapeutics, association score

## Abstract

Hanahan and Weinberg introduced the “hallmarks of cancer” and typified essential biological abilities acquired by human cancer. Since then, a growing understanding of hallmark principles associated with breast cancer has assisted knowledge-based therapeutics development; however, despite the rapidly increasing number of targeted therapeutics, enduring disease-free responses for most forms of breast cancer is rare. Invasion and metastasis are the most defining feature of breast cancer malignancy and the leading cause of patient mortality. Hence, we propose a modified hallmarks model adapted to breast cancer, in which invasion and metastasis are shifted to the center of attention, thereby emphasizing it as a potentially superior therapeutic target. Although the scientific community highly appreciates the importance of the invasion and metastasis hallmark, as can be demonstrated by the growing number of publications on breast cancer metastasis, very few clinical trials concentrate on testing anti-metastasis inhibitors and even fewer trials focus on inhibitors for breast cancer metastasis. Here, we discuss the obstacles of applying research on invasion and metastasis therapeutics into the clinic and present current developments that could provide a potential solution to this dilemma.

## 1. Introduction

Breast cancer is the most common neoplasm and the second leading cause of death in women [1,2].

It is a complex and highly heterogeneous disease, encompassing multiple tumor subtypes, each characterized by a different morphology, histopathological features and cellular markers, gene expression profiles, outcomes and response to systemic therapies [3,4,5]. Because of its complexity, immunohistochemical markers, together with clinicopathological criteria such as tumor size and grade, lymph node involvement and histological type, are commonly used for prognosis, recurrence prediction and therapeutic strategy selection [6,7].

Breast tumor subtypes are classically defined according to hormonal and growth factor responsiveness. The main clinically relevant receptors in this regard are estrogen receptor (ER), progesterone receptor (PR) and human epidermal growth factor receptor 2 (HER2). Breast tumors are thus divided into four main subtypes based on immunohistochemical properties and hormone receptor (HR) status [3]: (1) HR^+^/HER2^+^, tumors which are sensitive to either estrogen or progesterone and present HER2 receptors, (2) HR^+^\HER2^−^, tumors which are sensitive to either estrogen or progesterone but lack HER2 receptors, (3) HR^−^\HER2^+^, tumors which express HER2 receptors, but lack hormone receptors and (4) HR^−^\HER2^−^, also known as triple-negative breast cancer (TNBC).

In 2000, Weinberg and Hanahan defined six hallmarks of cancer: sustaining proliferative signaling, evading growth suppressors, resisting cell death, enabling replicative immortality, inducing angiogenesis and activating invasion and metastasis [8]. In 2011, the authors defined two additional emerging hallmarks, reprogramming of energy metabolism and evading immune destruction and two enabling hallmarks, genome instability and mutation and tumor-promoting inflammation [9]. Together, these constitute “the ten hallmarks of cancer”. Here, we discuss each of these ten hallmarks and their relevance to breast cancer.

## 2. The Hallmarks of Breast Cancer

### 2.1. Sustaining Proliferative Signaling

Normal tissues control the production and release of growth signals that regulate proper entry and progression through the cell cycle and regular tissue architecture and function. Cancer cells de-regulate these growth signals and, by doing so, acquire the capability to sustain proliferative signaling in several ways. For example, cancer cells can enhance autocrine stimulation [10,11] or paracrine stimulation [12] with stromal cells. They elevate cell surface receptors’ expression and accumulate activating mutations that lead to constitutive activation of cell surface receptors or their downstream signaling pathways.

Several key receptors and their downstream signaling pathways have emerged as critical drivers of breast cancer development and growth, as well as potential therapeutic targets. For example, growth factor receptor pathways, particularly receptor tyrosine kinases (RTKs), play a significant role in initiating proliferative and cell survival pathways that are normally tightly controlled [13,14]. Among these, the ErbB family of RTKs has been studied most extensively in breast cancer [15], but other RTKs such as the insulin-like growth factor 1 receptor (IGF1R) have also been explored [16,17].

*HER2* amplification or protein overexpression is found in 20–30% of invasive breast cancers and is associated with accelerated cell growth, proliferation and poor clinical outcomes [18,19]. One of the most common inhibitors of HER2 is trastuzumab, a monoclonal antibody that binds to the extracellular domain of HER2 and inhibits several major downstream signaling pathways that regulate tumor growth, such as the RAS/RAF/MEK/MAPK and the PI3K/AKT pathways [20,21]. In addition to activation by homodimerization, enhanced by overexpression of the receptor in breast cancer, HER2 can dimerize with other ErbB family members such as the epidermal growth factor receptor (EGFR). Indeed, targeting both receptors simultaneously can provide therapeutic synergy, which is achieved by using the tyrosine kinase inhibitor lapatinib. Lapatinib inhibits tyrosine phosphorylation and activation of both EGFR and HER2 and, in turn, inhibits activation of the pro-proliferative kinases ERK1/2 and AKT [20].

Elevated levels of insulin-like growth factor 1 (IGF1) and excessive IGF1R signaling have been implicated in an increased risk of breast cancer [22]. IGF1R inhibitors currently in use include neutralizing antibodies and tyrosine kinase inhibitors. It is now understood that extensive crosstalk of IGF1R downstream signaling pathways with other breast cancer signaling pathways exists. Moreover, up-regulation of IGF1R is used by breast cancer cells as a mechanism of resistance to HER2, EGFR and anti-estrogen inhibitors. These mechanisms suggest combining IGF1R inhibitors with other targeted therapies as an effective therapeutic strategy [20,23].

Determining tumor sensitivity to proliferative signaling is crucial in selecting appropriate breast cancer treatment. Indeed, several targeted therapies which aim to block breast tumor cell proliferation are currently being used at the clinic. Tamoxifen is an ER blocker that inhibits estrogen sensitivity and therefore is tailored for the ER^+^ subtypes, luminal A and ER^+^ luminal B. Trastuzumab, on the other hand, binds to HER2 and shows a highly beneficial effect in subtypes that overexpress HER2, such as HER2^+^ luminal B and HR^−^/HER2^+^. TNBC, which is the most aggressive breast cancer with the highest metastatic potential, has no targeted treatment at present and is usually treated by chemotherapy [2]. In addition, ER, PR and HER2, another surface marker worth mentioning is the androgen receptor (AR) [24,25]. AR has been shown to have some predictive value in breast cancer, with an overall mixed effect of increased survival and increased resistance to tamoxifen [26]. Several anti-androgenic drugs have been proposed as candidates for AR targeted treatment [26,27,28,29]. This new type of targeted treatment could be beneficial if proven effective, especially in otherwise unresponsive AR^+^/HER2^+^ and AR^+^/TNBC [26].

### 2.2. Genome Instability and Mutation

The success of the neoplastic process depends on high mutation rates of the genome of neoplastic cells. One way cancer cells increase their mutation rate is by disrupting one or several components of the genomic maintenance and repair machinery. Such alterations are selectively advantageous for tumor progression because they accelerate the rate at which pre-malignant cells can accumulate mutations and become cancerous [8,9]. Approximately ten percent of all breast cancer cases are related to genetic predisposition or family history, with a variance of geographical region and ethnicity [30]. Familial cancer predisposition is usually correlated with alterations in components of the DNA maintenance and repair machinery. The most common germline mutations associated with breast cancer are in breast cancer susceptibility genes 1 and 2 (BRCA1 and BRCA2), which encode proteins responsible for double-stranded DNA repair. Germinal mutations in these two genes correlate with a high rate of somatic gene mutations and subsequently a risk factor of breast and ovarian cancers [31]. In addition, mutations in BRCA1 are associated with the more aggressive, receptor-negative phenotype, whereas BRCA2 mutated breast cancers are predominantly hormone-sensitive. The latter, nevertheless, demonstrates significantly lower survival rates than BRCA^−^/HR^+^ breast cancer patients [32].

### 2.3. Resisting Cell Death

Disruption of the apoptotic process allows tumor cells to evade their intrinsic self-destruction mechanism. The apoptotic trigger is controlled by counter-balancing pro- and anti-apoptotic members of the B-cell lymphoma 2 (BCL2) family of regulatory proteins. Pro-apoptotic genes are typically known to enhance tumor growth and increase resistance to breast cancer therapy [33]. *BCL2*, a well-recognized prognostic factor, is an anti-apoptotic (pro-survival) gene. Specifically, BCL2 is an estrogen-responsive gene and therefore tends to be overexpressed in primary ER^+^ tumors [34]. The correlation of BCL2 with estrogen sensitivity may be the underlying reason for the relatively high survival rates of BCL2^+^ breast cancer patients. In other words, the overall better prognosis of ER^+^ tumors outweighs the prospective damage promoted by BCL2. In recent studies, BCL2 inhibitors have been suggested as a possible therapeutic approach to breast cancer, both due to their apoptosis-enabling effect and their potential synergistic effect with tamoxifen as an ER inhibitor. One such inhibiting drug, venetoclax, is a selective BCL2 inhibitor used to treat leukemia but has not yet been evaluated in solid tumors. It has now undergone a phase Ib clinical trial in breast cancer patients to determine safety preparation for further clinical trials [33]. Therefore, resistance to cell death is a possible effectual target for new breast cancer therapies.

### 2.4. Enabling Replicative Immortality

Telomers which protect the ends of chromosomes are essential for the capability of unlimited proliferation of cancer cells. Telomerase is almost absent in non-immortalized and normal cells but is highly expressed in most human cancer cells. Overexpression and high activation of telomerase in pre-malignant cells enable the maintenance of their telomeric DNA at lengths that are sufficient to avoid the triggering of senescence or apoptosis, leading to their immortalization and transformation into malignant cells. Telomerase is activated in 85% of all human cancers [35] and nearly all breast cancer cases [36].

Breast cancer cell telomeres are much shorter than those of normal cells [37], suggesting that tumor cells need to undergo several replication cycles before reaching a lower limit that induces telomerase activity and consequent replication immortality. Therefore, it is thought that telomerase inhibitors may serve as a viable anti-cancer treatment that will cause the tumor to enter senescence and die of old age. Furthermore, such an approach should be relatively safe, as telomerase activity is absent from most healthy cells [36]. However, no telomerase-targeting therapy has been approved so far for clinical use [38].

A recent study examined the inhibition of telomerase by small interference RNA (RNAi) combined with chemotherapy (doxorubicin) on breast cancer cell lines of three types: luminal A (MCF-7), TNBC (MDA-MB-468) and HER2^+^ (SKBR-3). The combined treatment resulted in an over 90% reduction in telomerase activity and a decrease of more than 60% in cell viability, demonstrating a cumulative effect of chemotherapy. These promising findings may offer a potential new strategy for the inhibition of breast cancer [39].

### 2.5. Evading Growth Suppressors

One of the mechanisms by which cancer cells maintain a high proliferation rate is by evading programs that negatively regulate cell proliferation. These programs, which exist in every normal cell, mainly depend on tumor suppressor genes. *TP53* is a prototypical tumor suppressor gene that governs a wide range of cellular functions such as cell proliferation, apoptosis, DNA repair, gene expression and metabolism. The TP53 protein receives input from stress and abnormality sensors within the cell and decides, based on the levels of DNA damage caused in the cell genome, whether to halt the cell cycle and enable repair or to trigger apoptosis in cases where excessive damage that cannot be repaired exists [40].

Inactivating mutations in *TP53* are observed in nearly 50% of all cancers and 25–30% of the basal-like subtype of breast cancer [41]. Moreover, women who carry a *TP53* germline mutation have an 85% chance of developing breast cancer by the age of 60 [42]. Thus, in addition to being a prognostic factor, the *TP53* mutation profile may influence the effectiveness of chemotherapy and hormonal therapy, which are used to treat breast cancer and are therefore examined before administration of such treatments [41,43,44,45].

### 2.6. Reprogramming Energy Metabolism

The uncontrolled cell proliferation that represents neoplastic disease involves, in addition to de-regulated control of cell proliferation, corresponding adjustments of energy metabolism to fuel cell growth and division. Thus, a common observation in neoplastic disease is a sharp increase in glucose consumption that accompanies a shift to anaerobic glycolysis instead of a more efficient aerobic mitochondria-mediated oxidative phosphorylation utilized by healthy nucleated cells. These changes are usually related to activation of RAS and MYC and inactivating mutations in *TP53* [46], enhancing the continuous proliferation of cancer cells.

In breast cancer, there is a difference in metabolic activity between the different subtypes, as both hormones and growth factors regulate glucose consumption and energy metabolism, leading to slightly different enzymatic profiles of ER^+^, HR^+^ and TNBC tumors [47]. Furthermore, blocking proliferation signaling, a preferred strategy for treating receptor-positive breast cancer subtypes, leads to downstream metabolic changes, further disrupting overall tumor growth [47].

### 2.7. Inducing Angiogenesis

Like normal tissues, tumors require nutrients and oxygen and the ability to evacuate metabolic waste and carbon dioxide. These needs are emphasized in tumors due to the rise in glucose consumption and the ever-increasing energy demands of the growing tumor. Tumor-associated neo-vasculature, generated by the process of angiogenesis, addresses these needs. Angiogenesis is a process that is transiently activated during embryogenesis and in normal physiological processes such as wound healing and the female reproductive cycle in the adult. During tumor progression, an angiogenic switch is activated and remains constitutively on, leading to continuous sprouting of new vessels which help to sustain expanding neoplastic growth [8,9].

Bevacizumab (Avastin) is a monoclonal antibody that targets the vascular endothelial growth factor (VEGF), a prototypical promoter of angiogenesis. Bevacizumab is used to treat many types of cancer, such as non-small-cell lung cancer, glioblastoma, renal cell carcinoma, ovarian cancer and cervical cancer [48]. Similarly, bevacizumab was approved by the federal drug and use administration (FDA) for use in metastatic breast cancer in 2008. However, this approval was reversed two years later due to a lack of supporting evidence from clinical observations and safety concerns [48,49]. The assessment of the FDA regarding the use of bevacizumab in breast cancer was considered controversial, especially in cases of HER2^−^ breast cancer and TNBC, which are highly resistant to standard breast cancer therapy [48]. Today, bevacizumab is used to treat breast cancer in Europe and other countries parallel to its continuous examination in clinical trials [48,49]. The efficacy variation of bevacizumab between breast cancer and other cancers may be explained by differences in the underlying angiogenesis-related mechanisms of these distinct cancer types.

### 2.8. Tumor Promoting Inflammation

It has long been recognized that some tumors are densely infiltrated by immune cells, thereby mirroring inflammatory conditions in non-neoplastic tissues. Such tumor-associated immune responses were initially thought to reflect an attempt of the immune system to eradicate tumors. However, it is now known that inflammation can also contribute to multiple hallmark capabilities by supplying bioactive molecules such as growth and survival factors, pro-angiogenic factors and extracellular matrix modifying enzymes to the tumor microenvironment, which supports tumor progression and metastasis. Inflammatory cells can also release reactive oxygen species mutagenic for nearby cancer cells, accelerating their genetic evolution towards malignancy [8,9].

### 2.9. Evading Immune Destruction

According to the immune surveillance theory, cells and tissues are constantly monitored by the immune system, which recognizes and eliminates most developing cancer cells and immature tumors that accidentally develop [50]. According to this logic, the tumors that succeed developing have somehow managed to avoid detection by the immune system or limit the extent of targeted killing by the immune system, thereby evading eradication. Immunogenic cancer cells may escape immune destruction by disabling components of the immune system addressed to eliminate them. They do so by secreting immunosuppressive factors and recruiting immunosuppressive cells such as regulatory T cells and myeloid-derived suppressor cells that can suppress the actions of cytotoxic lymphocytes [8,9].

Understanding the mechanisms used by cancer cells to evade immune destruction has led to the development of immunotherapy, which is based on stimulating a patient’s immune system to recognize and destroy cancer cells more effectively. An essential part of the immune system is its ability to keep itself from attacking normal cells in the body. To do so, it uses “checkpoints,” proteins on immune cells that need to be turned on or off to start an immune response. Breast cancer cells often use these checkpoints to avoid being attacked by the immune system. Therapies that target these checkpoint proteins assist in restoring the immune response against breast cancer cells. Today, immunotherapy is used to treat some types of breast cancer. Pembrolizumab (Keytruda) is a drug that targets programmed cell death 1 protein (PD-1), a protein on T cells that normally help keeps these cells from attacking other cells in the body. By blocking PD-1, this drug boosts the immune response against breast cancer cells and often leads to tumor shrinkage. Pembrolizumab is used in combination with chemotherapy to treat TNBC which expresses programmed cell death protein ligand 1 (PD-L1). Atezolizumab targets PD-L1, the ligand of PD-1, which is found on some tumor cells and immune cells. Blocking this protein can help boost the immune response against breast cancer cells and consequently shrink some tumors or slow their growth. Atezolizumab is used in combination with paclitaxel for advanced TNBC when the tumor expresses the PD-L1 protein [51].

The immunogenicity of breast cancer is heterogeneous and depends mainly on the specific subtype of breast cancer. Although ER^−^ and HER2^+^ tumors have shown evidence of immunogenicity, these types of inflammatory breast tumors are rare when compared to TNBC tumors, which are unique among breast cancer subtypes in having strong antigen expression and high stromal and tumor-infiltrating lymphocytes, parameters with a vital prognostic and predictive significance to immunotherapy and chemotherapy. Except for these immunogenic subtypes, breast tumors are usually considered immunologically silent or “cold” tumors, characterized by the presence of low mutation and neoantigen burden and few tumor-infiltrating lymphocytes. In addition, resistance to cancer immunotherapies often develops in breast cancer due to alternative immunosuppressive checkpoint pathways. Therefore, despite the use of combination immunotherapy in metastatic TNBC patients today, the immunologic treatment of breast cancer remains a significant challenge [52].

### 2.10. Invasion and Metastasis

Metastatic dissemination of tumor cells, also known as the invasion-metastasis cascade [9], is a multi-step process that starts with local invasion of tumor cells into the surrounding stroma, followed by intravasation into the vasculature, endurance of shear stress in the vasculature, extravasation to distant sites and organs and, finally, adaptation to a new microenvironment and the formation of micro-metastases that will develop into a detectable secondary tumor. Metastatic dissemination of breast cancer cells from the primary tumor and their spread into distant sites in the body is the leading cause of mortality in breast cancer patients. Moreover, while primary breast tumors can arise from various genetic causes and hallmarks, metastasis is a common stage and should serve as a primary target for therapeutic intervention. Current therapeutic standards for breast cancer involve surgical resection of the primary tumor accompanied by radiation therapy or chemotherapy. Cytotoxic drugs and hormone-blocking therapeutics are often used in breast cancer; however, they do not prevent metastasis as they mainly affect proliferation, cell cycle and apoptosis. Despite the importance and centrality of the invasion and metastasis hallmark in breast cancer, no treatment to permanently eradicate metastatic breast cancer exists at present.

Fundamental understanding of the cancer hallmarks has facilitated the development of knowledge-based therapeutics. Regardless of the rapidly growing number of targeted therapies, their effectiveness is usually transient and prolonged progression-free survival and remedy are rare; the chances of survival usually increase if the tumor is detected early and surgically excised [53]. Two major reasons explain the failure of current targeted therapies to cure breast cancer patients. First, the metastatic process, which is the leading cause of disease recurrence and mortality in breast cancer patients, can arise during the early stages of pre-malignant cancer, while current therapeutics target all other hallmarks of breast cancer excluding invasion and metastasis. Second, breast cancer cells undergo Darwinian evolution and natural selection in response to therapies that target hallmarks such as proliferation, apoptosis and cell survival. This process leads to the development of tumor cells resistant to therapy. Notably, while treatments for most cancer hallmarks are currently available, no cure for invasion and metastasis in general and breast cancer in particular currently exists.

Gene expression profiling of breast tumors has yielded signatures from which prognostic tests could be derived to facilitate clinical and therapeutic decision-making in breast cancer patients. Interestingly, although intended to predict the metastatic potential of breast tumors, most of these tests gain predictive and prognostic power from the quantification of genes that are involved in proliferation and are efficient for only specific subtypes of breast cancer [54]. Using the needle collection technique, which enables isolation of the invasive population of cancer cells from a tumor, it was previously demonstrated that proliferation and cell cycle genes are down-regulated, while the expression of motility and invasion and DNA-repair genes is up-regulated in invasive breast cancer cells within the primary tumor [55]. Today’s predictive tests are based on whole tumor tissue, which includes a heterogeneous population of tumor cells and various stromal cells. Considering that in every given moment only a small fraction of cancer cells invades the surrounding tissue and intravasates into blood vessels and due to the transient, rare and short-lived nature of the metastatic process, gene expression changes which regulate invasiveness and metastasis in breast tumor cells within the primary tumor would be missed by profiling whole tumor chunks.

Within primary breast tumors, tumor cell intravasation occurs at micro-anatomical doorways on blood vessels of the tumors, called Tumor Micro Environment of Metastasis (TMEM). Each functional TMEM doorway comprises three different cell types in direct and stable physical contact: a tumor cell highly expressing the actin-regulatory protein Mammalian-enabled (MENA), a tumor-associated macrophage expressing high levels of perivascular tyrosine kinase with immunoglobulin-like loops and epidermal growth factor homology domains 2 (TIE2) and VEGF (TIE2^hi^/VEGF^hi^) macrophage and an angiopoietin 2 (ANG2) secreting endothelial cell [56,57]. TMEM doorways have been identified in mouse and human mammary carcinomas and their density correlates with metastatic outcomes in breast cancer patients [58,59,60]. Because intravasation of tumor cells into blood vessels is a critical step in the systemic spread of tumor cells and a general mechanism that metastatic cells use, which does not directly depend on changes in gene expression or natural selection within the primary tumor, TMEM may be preferable as a method for predicting the metastatic potential of breast tumor cells.

## 3. Scientific Research and Therapeutic Applications

**Figure 1:**
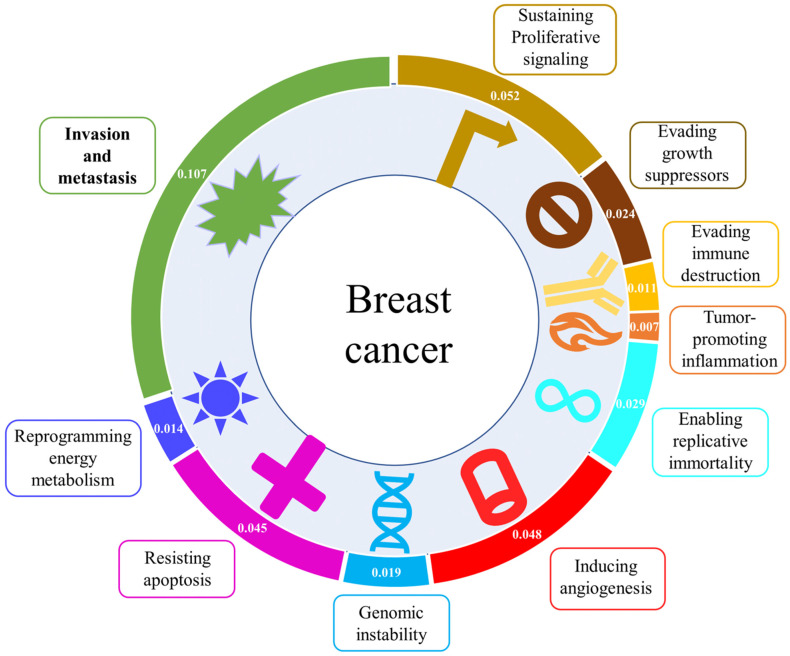
Outline of the ten hallmarks of cancer with PubMed association score for each hallmark keyword with breast cancer. The association score of each of the hallmarks, represented by one of the keywords: “proliferation”, “growth”, “immune”, “inflammation”, “immortality”, “angiogenesis”, “mutation”, “apoptosis”, “metabolism”, “metastasis”, AND “breast cancer” was calculated using PubMed citations. The number of citations for each hallmark with breast cancer was then divided by the number of each query term alone according to the following equation: association score = (citations of “keyword” for each hallmark AND “breast cancer”)/(citations of “keyword” for each hallmark). Note that the association score of invasion and metastasis is significantly higher than the other hallmarks, suggesting that this hallmark is highly explored and should be considered a prime therapeutic target.

**Figure 2:**
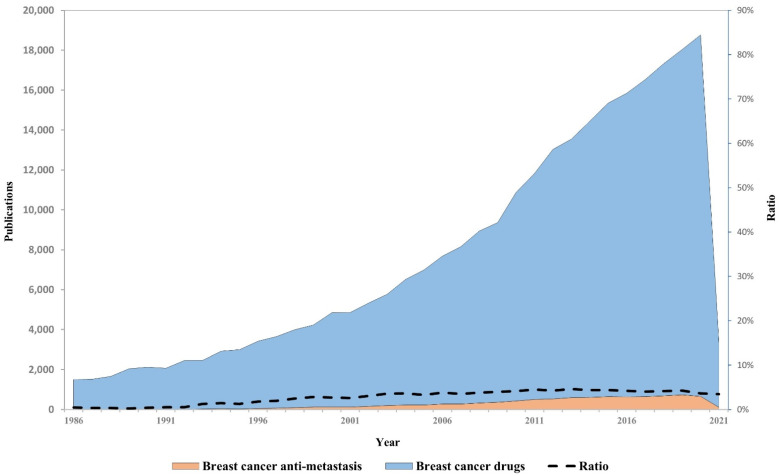
Annual publications associated with breast cancer drugs versus breast cancer anti-metastatic drugs. Total annual publications among breast cancer-related research in the years 1986−2021 as obtained by PubMed search. The search query used to retrieve drug-associated publications was: (“drug”|“drugs”|” treatment”|“treatments”|“therapy”|“therapies”) AND (“breast”). The search query used to retrieve antimetastatic-associated publications was: (“anti-metastatic”|“antimetastatic”|“anti-metastasis”|“antimetastasis”|“anti-invasive”|“anti-invasion”|“anti-migratory”|“anti-migration”|“anti-degradation”|“antiproteolytic”|“antiproteolysis”|“anti-MMP”|“anti-matrix metalloproteinase”|“metastasis prevention”|“metastatic prevention”|“anti-invadopodia”) OR (“invasion”|“invadopodia”|“migration”|“degradation”|“proteolysis”|“proteolytic”|“MMP”|“matrix metalloproteinase”) AND (“inhibitor”|“inhibitors”|“antagonist”|“antagonists”) AND (“breast”). The ratio between the queries is represented by a dashed line.

To gain an insight into research efforts invested on each of the hallmarks, we analyzed the association between each of the ten hallmarks and relevant scientific research publications. As demonstrated in Figure 1, the invasion and metastasis hallmark is associated with the most significant number of publications (association score 0.107), implying that this hallmark is highly explored. Not surprisingly, the second highly studied hallmark is sustaining proliferative signaling (association score 0.052), demonstrating that significant effort is still invested in the proliferation of the primary tumor. We next compared research publications related to anti-metastasis inhibitors versus all research publications associated with breast cancer therapeutics. Surprisingly, only a small fraction (<3%) of publications was related to anti-metastasis inhibitors, while most publications on breast cancer therapeutics address the other hallmarks (Figure 2). Moreover, although the importance of the invasion and metastasis hallmark is highly appreciated in the scientific community and as can be demonstrated by the constantly growing number of publications on breast cancer metastasis and therapeutics, very few clinical trials concentrate on testing anti-metastasis inhibitors at present and even less of them focus on inhibitors of breast cancer metastasis. These data suggest that while the invasion and metastasis hallmark is highly appreciated and widely researched in the laboratory, a significant gap exists between research performed in the laboratory and its clinical and therapeutic applications.

Translating research findings in invasion and metastasis into clinical applications is challenging for two major reasons. First, tumor size, which is affected by all other hallmarks of cancer besides invasion and metastasis, is considered an indicator for cancer progression mainly because it is a measurable endpoint and is constantly used in clinical trials. However, tumor size does not usually correlate with the invasion and metastasis potential of tumor cells and can sometimes even have an opposite correlation [61]. Second, as metastatic dissemination is a prolonged process, which can sometimes even appear after a decade or two after initial diagnosis and treatment, considerations such as time frame of the experiment, treatment period with the inhibitor which is meant to prevent metastasis, the starting point of the experiment and selection of correct experimental groups, become relevant. Several recent developments could offer a solution to this dilemma. First, an FDA draft guidance defined metastasis-free survival (MFS) as a valid endpoint in clinical trials was recently published [62]. Defining MFS as an endpoint instead of overall survival enables earlier evaluation of prognostic criteria in general and metastatic dissemination. Second, it was previously published that cytotoxic treatment of breast cancer such as neoadjuvant therapy can potentially induce cell invasiveness and metastatic dissemination due to its ability to result in cell damage and wound healing-like inflammatory processes which recruit immune cells that are manipulated by tumor cells to become pro-metastatic. Performing clinical trials with anti-metastatic drugs on breast cancer patients who receive neoadjuvant therapy could shorten the experiment time and enable faster evaluation of results. Indeed, several clinical trials which use the neoadjuvant setup are currently being performed with the Tie2 inhibitor rebastinib, which is tested for its ability to inhibit neoadjuvant-induced breast cancer metastasis.

## 4. Concluding Remarks

Even though the hallmarks model excellently illustrates what goes wrong in breast cancer cells, it fails to interrogate the hierarchy of the hallmarks in the broader aspect of therapeutic approaches. Being the most defining characteristic of malignancy, the invasion and metastasis hallmark should be placed in the center of attention and considered a potentially superior therapeutic target. Our analysis demonstrates that, while it is well recognized that invasion and metastasis is the most important hallmark of breast cancer, its prevention receives very little attention in the literature compared to preventing other hallmarks in primary or secondary breast tumors. These observations justify the necessity of shifting both scientific and clinical attention towards the invasion and metastasis hallmark as a potential therapeutic target in breast cancer. With new developments such as defining MFS as an endpoint in clinical trials and recognizing neoadjuvant treatment as a possible accelerator of breast cancer metastasis, this strategy becomes feasible. A successful application of this concept will depend on a collaborative effort of researchers, physicians and pharmaceutical scientists.

## Data Availability

Not applicable.
